# Ibuprofen-Induced Hemolytic Anemia

**DOI:** 10.1155/2013/142865

**Published:** 2013-04-21

**Authors:** Aram Barbaryan, Chioma Iyinagoro, Nwabundo Nwankwo, Alaa M. Ali, Raya Saba, Shawn G. Kwatra, Nasir Hussain, Chukwuemeka C. Uzoka, Suartcha Prueksaritanond, Aibek E. Mirrakhimov

**Affiliations:** Department of Internal Medicine, Saint Joseph Hospital, 2900 N. Lake Shore, Chicago, IL 60657, USA

## Abstract

Drug-induced immune hemolytic anemia is a rare condition with an incidence of 1 per million of the population. We report the case of a 36-year-old female who presented to the emergency department complaining of shortness of breath and dark colored urine. Physical examination was significant for pale mucous membranes. The patient reported using ibuprofen for a few days prior to presentation. Complete blood count performed before starting ibuprofen revealed normal platelets and hemoglobin values. On admission, the patient had evidence of hemolytic anemia with hemoglobin of 4.9 g/dL, hematocrit of 14.2%, lactate dehydrogenase 435 IU/L, and reticulocytosis 23.2%. Further testing ruled out autoimmune disease, lymphoma, and leukemia as etiologies for the patient's new onset hemolytic anemia. Ibuprofen was immediately stopped with a gradual hematologic recovery within 3 days.

## 1. Introduction

Drug-induced immune hemolytic anemia (DIIHA) is a serious condition that can be a rare side effect of commonly used over-the-counter medications. The incidence of DIIHA is estimated to be 1 per million of the population. While no explicit data exist regarding the incidence of ibuprofen induced hemolysis, various case series have found that NSAIDs compromise less than 15% of cases; the majority of cases are caused by beta-lactamase antibiotics (e.g., cephalosporins and penicillins). Timely recognition of this condition along with discontinuation of the offending agent is paramount in treating its potentially fatal complications [1–3].

## 2. Case Presentation 

A 36-year-old healthy caucasian female presented to the emergency department complaining of 1-day duration of shortness of breath. The only medication that she was taking was ibuprofen, which she started to take twice daily one week before presentation for tension headaches. The patient was found to have regular sinus tachycardia of 127 beats per minute. Subsequent physical examination revealed jugular venous distention and pale mucous membranes with no jaundice, lymphadenopathy, or organomegaly. No obvious source of bleeding was identified.

**Figure 1:**
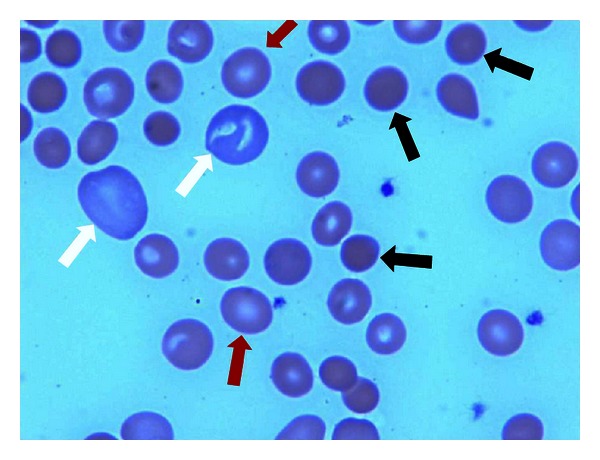
Peripheral blood smear shows microspherocytes (black arrows), polychromasia (white arrows), and normal looking red blood cells with central pallor (red arrows).

Upon initial workup, she was found to be severely anemic with a hemoglobin of 4.9 (normal range 12–15 g/dL), hematocrit of 14.2 (normal range 36%–47%), MCV 98.8 (normal range 80–100 fL), and RDW of 24.6 (normal range 11%–14.5%). Anemia workup was initiated and was significant for an elevated lactate dehydrogenase (LDH) 435 (normal range 135–214 IU/L), reticulocytosis 23.2 (normal range 0.5%–2.8%), and decreased haptoglobin <6 (normal range 36–195 mg/dL). Stool test for occult blood was negative. Type and screen were positive for antibodies and Direct Antiglobulin Test (DAT) was reactive with anti-IgG and anti-C3 antibodies. Coombs test elution was also found to be positive. Biochemical tests except for bilirubin 2.7 (normal range 0.0–1.0 mg/dL) were unremarkable. The patient's peripheral blood smear demonstrated an abundance of spherocytes and polychromasia (Figure 1). No abnormalities were noted among white blood cells and platelets. Antinuclear antibodies (dsDNA, Chromatin, Ribosomal P, SS/A, SS/B, Sm, SmRNP, RNP, Scl 70, Jo 1, and Centromere B), rheumatoid factor (RF) and CT scan of chest, abdomen, and pelvis were all negative as part of the workup for autoimmune hemolytic anemia.

Patient was transfused three units of packed red blood cells (PRBC), after which time her hemoglobin increased from 4.9 to 6.9 g/dL. Ibuprofen was stopped immediately upon presentation and a maintenance dose of prednisone was initiated along with aggressive hydration therapy. She responded well to the above mentioned measures and had an uneventful recovery. Patient was examined a few weeks later which showed complete hematologic recovery.

## 3. Discussion

Drug-induced immune hemolytic anemia (DIIHA) is a rare condition, affecting approximately 1 per million of the population. As a comparison, drug-induced thrombocytopenia and neutropenia have an incidence of 10–18 cases per million and 2–15 cases per million, respectively [4, 5]. DIIHA can be further classified by dividing the drug into whether or not antibodies to the drug are present: drug dependent (DDAB) and drug independent (DIAB). DDAB shows activity only in the presence of the drug; DIAB has activity in the absence of drug [6]. Most cases of DIIHA are caused by DDABs. Usually direct antigen testing (DAT), which is also known as the direct Coombs test, is used to diagnose DIIHA [6]. In this test washed RBCs are mixed with antiserum or monoclonal antibodies prepared against IgG and a third component of complement C3d [7]. It is positive almost in all cases of DIIHA, although some rare cases of negative DAT can be occasionally seen [2]. If DAT is positive, then elution test should be performed to characterize antibodies. In case of DDAB elution test is negative since drug is not present in vitro testing. DIIHA is less frequently mediated by DIAB which is almost identical to warm autoimmune hemolytic anemia (WAIHA). In this case both DAT and elution are positive. The only way to differentiate between DIAB and WAIHA is to stop the causative agent and observe the hematologic response [6]. Usually it takes a few weeks to reach hematological remission, meanwhile serological remission (when Combs test becomes negative) might take a few months [2]. Treatment of DDAB is discontinuation of an offending drug. In the case of DIAB, steroids should be also added besides culprit drug discontinuation [6].

The main diagnostic entities to be considered in the differential diagnosis of drug-induced immune hemolytic anemia (DIIHA) include different causes of warm autoimmune hemolytic anemia (WAIHA), since they are characterized by IgG antibodies that react with red blood cell antigens at body temperatures. The main causes of WAIHA are idiopathic WAIHA, autoimmune and connective tissue diseases (especially systemic lupus erythematosus, scleroderma, dermatomyositis, ulcerative colitis, and rheumatoid arthritis), lymphoma, chronic lymphocytic leukemia, and prior organ transplantation [8–13]. DIIHA is most commonly confused with idiopathic WAIHA, which is more common [2]. An elution test can be useful in differentiating DIIHA from WAIHA. In the case of WAIHA, both DAT and elution tests are positive. In rare cases of DDAB and in almost all cases of DIAB, the elution test can be positive too. In this scenario the only way to confirm the diagnosis of DIIHA is documentation of complete hematologic and serologic recovery after discontinuing the offending medicine [6]. 

In conclusion, commonly used over-the-counter medications can have rare but serious side effects. Because of their rarity, drug-induced hemolytic reactions are less well investigated compared with drug-induced thrombocytopenias and granulocytopenias. Timely recognition of the causative agent cannot be underestimated since failure to do so can result in continuation of the offending drug and worsening of the patient's hemolytic anemia. In addition to discontinuation of the drug and monitoring for hematologic recovery, steroids occasionally might also be needed.
